# Application of Exogenous Ethylene Inhibits Postharvest Peel Browning of ‘Huangguan’ Pear

**DOI:** 10.3389/fpls.2016.02029

**Published:** 2017-01-18

**Authors:** Yurong Ma, Mengnan Yang, Jingjing Wang, Cai-Zhong Jiang, Qingguo Wang

**Affiliations:** ^1^Postharvest Laboratory, College of Food Science and Engineering, Shandong Agricultural UniversityTai’an, China; ^2^Crops Pathology and Genetics Research Unit, United States Department of Agriculture-Agricultural Research Service, DavisCA, USA; ^3^Department of Plant Sciences, University of California, DavisDavis, CA, USA

**Keywords:** ‘Huangguan’ pear, browning disorder, exogenous ethylene, antioxidant enzymes, total phenolics

## Abstract

Peel browning disorder has an enormous impact on the exterior quality of ‘Huangguan’ pear whereas the underlying mechanism is still unclear. Although different methods have been applied for inhibiting the peel browning of ‘Huangguan’ pear, there are numerous issues associated with these approaches, such as time cost, efficacy, safety and stability. In this study, to develop a rapid, efficient and safe way to protect ‘Huangguan’ pear from skin browning, the effect of exogenous ethylene on peel browning of pear fruits stored at 0°C was evaluated. Results showed that ethylene treatments at 0.70–1.28 μL/L significantly decreased the browning rate and browning index from 73.80% and 0.30 to 6.80% and 0.02 after 20 days storage at 0°C, respectively, whereas ethylene treatments at 5 μL/L completely inhibited the occurrence of browning. In addition, ethylene treatments at 5 μL/L decreased the electrolyte leakage and respiration rate, delayed the loss of total phenolic compounds. Furthermore, ethylene (5 μL/L) treatment significantly enhanced the activity of catalase (CAT), ascorbate peroxidase (APX) and superoxide dismutase (SOD) and increased the 1, 1-diphenyl-2-picrylhydrazyl inhibition rate, but inhibited the activity of polyphenol oxidase (PPO) and peroxidase (POD). Our data revealed that ethylene prevented the peel browning through improving antioxidant enzymes (CAT, APX and SOD) activities and reducing PPO activity, electrolyte leakage rate and respiration rate. This study demonstrates that exogenous ethylene application may provide a safe and effective alternative method for controlling browning, and contributes to the understanding of peel browning of ‘Huangguan’ pear.

## Introduction

Huangguan pear (*Pyrus bretschneideri* Rehd cv. Huangguan) is a new cultivar with comprehensive qualities and widely planted in northern China (Wang, 1998). However, a surface brown disorder in the peel (also known as chicken-claw disease by farmers in China) often occurs before harvest or during early stage of storage. Symptom in the affected area of the peel is lightly brown at first, and then becomes darker as the disorder progresses. The disorder usually only affects the peel of fruits but not the flesh. However, the surface brown disorder often seriously impacts on the exterior quality of ‘Huangguan’ pear and causes enormous economic loss.

Browning disorder of pears is affected by both preharvest factors (such as picking date, maturity, fruit size and kind of fruit-bags) and postharvest factors (such as the duration of cooling period, the storage temperature and the CO_2_ and O_2_ concentrations; Lammertyn et al., 2000; Guan et al., 2005, 2008; Galvis-Sánchez et al., 2006; Wang and Wang, 2011). For example, pears with higher content of chlorogenic acid, a dominant phenolic acid in ‘Huangguan’ pears, are prone to browning (Franck et al., 2007; Kou et al., 2015). Moreover, the occurrence of surface browning in ‘Huangguan’ pear was also reported to be related to the Ca^2+^ deficiency and the cellular Ca^2+^ distribution in skin tissues (Dong et al., 2015). In addition, heart browning and flesh browning of ‘Rocha’ pears are increased by the combination of 2 kPa O_2_ + 5 kPa CO_2_ (Galvis-Sánchez et al., 2006).

A number of different approaches such as slow cooling, methyl jasmonate, cold conditioning, 1-MCP and CaCl_2_ are being tried to reduce the incidence of peel browning of pears (Wang and Wang, 2011; Xing et al., 2013). Methyl jasmonate can effectively inhibit the peel browning of ‘Huangguan’ pear when cooled rapidly (Xing et al., 2013). A cold-conditioning at appropriate temperature (8–9°C) before cold storage (0°C) significantly inhibits the peel and core browning as well as reduces the accumulation of ethanol during storage and shelf life, maintaining the high edible quality of ‘Huangguan’ pear (Wang and Wang, 2011). Compared with control, treatments with 1-MCP, CaCl_2_ and 1-MCP + CaCl_2_ dramatically reduces the skin browning of ‘Huanguan’ pear (Gong et al., 2010).

Peel browning of fruits appears to be related to the damage of membrane integrity (Kou et al., 2015). The antioxidant enzymes such as superoxide dismutase (SOD), ascorbate peroxidase (APX), catalase (CAT) and peroxidase (POD) are believed participating in the browning of fruits and vegetables (Duan et al., 2011; Kou et al., 2015). These enzymes could protect the integrity of membrane from damage by scavenging H_2_O_2_, superoxide and other free radicals. Dipping with CaCl_2_ and pullulan reduces the incidence of brown spots of ‘Huangguan’ pear by decreasing the activity of polyphenol oxidase (PPO) and POD and increasing the activity of CAT and SOD (Kou et al., 2015). Application of pure oxygen induces the antioxidant enzymes (SOD, APX and CAT) activity in lichi fruit, thereby maintaining the membrane integrity and inhibiting the pericarp browning (Duan et al., 2011).

Though different methods have been applied for inhibiting the peel browning of ‘Huangguan’ pear, there are numerous issues associating with the approaches, such as time-consuming, higher cost, efficacy, safety and the stability of the efficiency. For example, slow cooling is commercially applied for the inhibition of peel browning. However, this cooling process is time-consuming. Moreover, after slow cooling, fruits show a higher rot rate and withered stalk rate (Wang and Wang, 2011). Thereby, developing a rapid, efficient and safe way to protect ‘Huangguan’ pear from skin browning is urgent and essential.

As a plant hormone, ethylene is believed to be responsible for the ripening and senescence of fruits and vegetables after harvest. However, some positive effects of ethylene on maintaining the quality of fruits are also reported (Zhou et al., 2001; Candan et al., 2008; Malerba et al., 2010; Lafuente et al., 2014). Ethylene plays important roles in protecting plants from various stresses. Zhou et al. (2001) found that the presence of ethylene during cold storage alleviated the woolliness of nectarines, a chilling injury phenomenon. Furthermore, ethylene conditioning at 12°C extended the shelf life of chilling injury sensitive and non-chilling peel pitting (NCPP) sensitive oranges. Moreover, ethylene conditioning prevented the initial decrease in flavonoid content and reduced the calyx abscission and NCPP (Lafuente et al., 2014).

Although application of ethylene has been well documented in various fruits, the effect of ethylene on the browning and antioxidant capacity in ‘Huangguan’ pear has not been reported. Thereby, the effect of ethylene on peel browning of ‘Huangguan’ pear was investigated. In order to understand the possible mechanism of browning, changes of total phenolics, the activity of PPO and antioxidant enzymes (POD, SOD, CAT and APX) during storage were also examined. Results of this study could benefit to the understanding of peel browning disorder of ‘Huangguan’ pear, and could also provide a safe and effective way for controlling pear peel browning for the industry.

## Materials and Methods

### Materials

Pears with optimum commercial maturity (based on soluble solid and firmness) were harvested in the harvest season from commercial orchards located in Jinzhou city and Gaocheng city, Hebei Province, China. After harvest, pears were immediately transported to the laboratory at Shandong Agricultural University (Tai’an, China). Fruits with similar size, maturity and without physical injury or infection were selected and randomly divided into groups for further use.

Slow-released ethephon (5% solid ethephon powder/sachet, 0.3 g/sachet) and 1-MCP (0.045% 1-MCP cyclodextrin powder, 0.4 g/sachet) were supplied by Shandong Yingyangyuan Food Technology Co., Ltd (Shandong, China).

### Ethylene Treatment

To investigate the effect of ethylene and 1-MCP on peel browning rate, pears harvested from Gaocheng city (Hebei Province, China) in 2012 were used. The fruits were packed in boxes with plastic liner, and 15 boxes of pears were randomly divided into five groups (three boxes of fruits for each group) with a total of 144 fruits (48 fruits per box as one repeat with three repeats, 48^∗^3 = 144 fruits). The fruits immediately stored at 0°C served as control. For air, 1-MCP, ethylene, and 1-MCP + ethylene treatments, fruits were treated by air (served as air control), slow-released 1-MCP (a sachet/box), slow-released ethylene (a sachet/box), and slow-released 1-MCP (a sachet/box) and ethylene (a sachet/box) at 20°C for 8 h, then stored at 0°C for 30 days. During the 8 h treatment, the concentrations of ethylene and 1-MCP were detected at 0.70–1.28 and 1 μL/L, respectively. On sampling day, fruits were taken from each treatment (48 fruits per repeat with three biological repeats, total of 144 fruits).

Effect of ethylene on physiological functions of ‘Huangguan’ pear was investigated with fresh pears harvested from Jinzhou city (Hebei Province, China) in 2014. Pears were randomly divided into three groups with 168 fruits per group (eight fruits per repeat for each sampling day, 7 sampling days, three repeats) and placed in the containers as mentioned above. For control, pears were packed with plastic foam sleaves, placed into trays overwrapped with plastic film, and immediately stored at 0°C (rapid cooling). For ethylene treatment, ethylene (10000 μL/L, ethylene/nitrogen) was injected into the sealed containers, maintaining the final concentration of ethylene (5 μL/L) at 20°C for 8 h. For air treatment, air with equal volume to ethylene was injected into the sealed container. Treatments were carried out at 20°C. After treatment, the pears were repacked with plastic foam sleeves, placed into trays wrapped with plastic film and stored at 0°C for 30 days. In addition, during cold storage, 24 fruits from each group (eight fruits per repeat with three biological repeats) were sampled on 0, 5, 10, 15, 20, 25, and 30 days, hand-peeled and manually cut into slices, and immediately frozen in liquid nitrogen. Samples (peel and pulp) were ground to fine powder in a mill and stored at -80°C for further analysis.

Prior to above experiments, a preliminary test was conducted to investigate the influence of ethylene concentration on peel browning. In this test, fruits harvested from Gaocheng city, Hebei Province, China, in 2012, were treated by 0, 5, and 50 μL/L ethylene at 20°C for 8 h as mentioned above (40 fruits per repeat with 3 replicates per treatment, 40^∗^3 = 120 fruits/treatment). Fruits immediately stored at 0°C were served as control (rapid cooling). Peel browning index and browning rate were determined 20 and 30 days post storage.

To examine long-term effects of ethylene treatments on the fruit quality, the firmness, titratable acidity (TA) and total soluble solids (TSS) were measured using the pears harvested in 2013. For detailed information, see **Supplementary Table S1**.

### Evaluation of Brown Spot Disorder

The degree of brown spot disorder was evaluated by the method previously described by Xing et al. (2013). This method was scored visually by the percentage of pear fruit surface covered by spots, with a scale from 0 to 4: 0 for no browning, 1 for 1–10%, 2 for 11–20%, 3 for 21–40% and 4 for 41–100%. Disorder index was calculated based on the formula of (fruit number × scale)/[total fruit number × 4 (the severest scale)].

### Electrolyte Leakage Assessment

Electrolyte leakage rate was determined using the method described by Liu et al. (2013) with a slight modification. Twelve disks were collected from eight fruits with a cork-borer (diameter 8 mm) and washed in distilled water three times. The disks were soaked in a glass tube containing 20 mL distilled water and incubated in a water bath shaker at 25°C for 2 h. The initial electric conductivity (C_0_) was measured using a conductivity meter. Then the glass tube was boiled for 30 min, cooled in room temperature and total electric conductivity (C_1_) was taken. Three biological replications were used for each treatment. Electrolyte leakage rate was calculated using the following equation: electrolyte leakage (%) = (C_0_/C_1_) × 100%.

### Respiration Rate Assessment

For each treatment, fruits were sealed in 4.5 L gas-tight jars at 0°C for 24 h. Gas samples were taken and then CO_2_ concentration was measured using a gas analyser (PBI-940437B). Three biological replications were used with six fruits in each treatment (six fruits per repeat). The respiration rate was expressed as L/(kg⋅h).

### Determination of Total Phenolics

The amount of total phenolics in pear peel was determined according to the Folin–Ciocalteu method described by Singleton and Rossi (1965). Two grams frozen pear peel tissues were extracted with 20 mL of 70% acetone and kept for 3 h in the dark. The extracts were centrifuged at 10000 × *g* for 20 min at 4°C. A 0.5 mL aliquot of supernatant was added to the reaction mixture (0.5 mL Folin–Ciocalteu and 0.5 mL of 10% Na_2_CO_3_). Then the mixture was diluted with distilled water to 10 mL and incubated in a water bath shaker at 25°C for 2 h. The absorbance was measured at 765 nm. The content of total phenolics was calculated from a standard curve developed with gallic acid and expressed as milligram gallic acid per gram fresh tissue (mg/g FW).

### Enzyme Assays

Polyphenol oxidase activity was assayed as previously described by Huang et al. (2009) with a slight modification. Peel (4 g) was extracted with 20 mL of 0.1 mol/L sodium phosphate buffer (pH 6.8) containing 5% polyvinylpolypyrrolidone (PVPP). Then the sample was homogenized and centrifuged at 10000 × *g* for 20 min at 4°C. The supernatant was used for analysis of PPO activity as well as POD activity (see below). Supernatants (0.4 mL) were mixed with 1 mL catechol (0.1 mol/L) and 2 mL of sodium phosphate buffer (0.1 mol/L, pH 6.8). The change in absorbance at 420 nm was measured every 30 s for 5 min. One unit of PPO activity was defined as the increase of 0.01 in absorbance per min under assay conditions. PPO activity was expressed as U/g FW.

The POD activity was determined according to Jiang et al. (2002) with some modifications. The reaction mixture contained 500 μL supernatant (see above), 2 mL 0.06% guaiacol in 0.1 mol/L sodium phosphate buffer (pH 6.0) and 1 mL 0.04% H_2_O_2_. One unit of POD activity was expressed as the change of 0.01 in absorbance at 470 nm per min. The result of POD was expressed as U/g FW.

To determine CAT activity, three grams of frozen tissue were ground with 10 mL sodium phosphate buffer (0.1 mol/L, pH 7.0) containing 5% PVPP. Then the sample was homogenized and centrifuged at 10000 × *g* for 20 min at 4°C. CAT activity was measured using the method of Beers and Sizer (1952) with some modification. 500 μL of supernatant was mixed with 2 mL sodium phosphate buffer (pH 7.0) and 500 μL H_2_O_2_ (0.1 mol/L). The absorbance was recorded every 30 s for 3 min. One unit of CAT activity was defined as the change of 0.01 in absorbance at 240 nm per min. The CAT activity was expressed as U/g FW.

Ascorbate peroxidase was determined according to Chen and Asada (1989) with modifications. APX was extracted from 3 g of the frozen pear peel tissue with 10 mL sodium phosphate buffer (50 mmol/L, pH 7.0, containing 0.1 mmol/L EDTA-Na2 and 5% PVPP) at 4°C. The homogenate was centrifuged at 10000 × *g* for 20 min at 4°C and the supernatant was used for the APX assay. Then, 500 μL supernatant was added to 3 mL reaction mixture containing 50 mmol/L sodium phosphate buffer (pH 7.0), 0.5 mmol/L ascorbate (extinction coefficient, 2.8 mM^-1^ cm^-1^) and 0.1 mmol/L H_2_O_2_. The absorbance of mixture was measured at 290 nm every 10 s for 1 min. One unit of APX was defined as the decrease of 0.01 in absorbance per min at assay conditions. The APX activity was expressed as U/g FW.

Superoxide dismutase was extracted from 3 g of the frozen pear peel tissue with 10 mL sodium phosphate buffer (50 mmol/L, containing 5% PVPP, pH 7.8) at 4°C. The homogenate was centrifuged at 10000 × *g* for 20 min at 4°C and the supernatant was used for the SOD assay. Then 500 μL enzyme extract was mixed with 1 mL sodium phosphate buffer (50 mmol/L, pH 7.8), 0.5 mL methionine (13 mmol/L), 0.5 mL nitroblue tetrazolium (NBT, 75 μmol/L), 10 μmol/L EDTA-Na_2_ and 2 μmol/L riboflavin. The mixtures were illuminated under 4000 Lux light for 15 min at 25°C (Li et al., 2013). The absorbance was determined spectrophotometrically at 560 nm. Non-illuminated solutions held in the dark served as a control. One unit of SOD activity was defined as the amount of enzyme that gave half-maximal inhibition of NBT reduction. The SOD activity was expressed as U/g FW.

### 1, 1-diphenyl-2-picrylhydrazyl (DPPH) Radical Scavenging Activity

The extraction method for 1, 1-diphenyl-2-picrylhydrazyl (DPPH) assay was used according to Gou et al. (2010). Three grams of the frozen pear peel was mixed with 12 mL of 95% ethanol and ultrasonically shaken at 50°C for 30 min. Then, the homogenate was centrifuged at 10000 × *g* for 10 min, and 2 mL supernate was added to 2 mL DPPH methanolic solution (0.2 mmol/L). After incubation for 30 min at room temperature in the dark, the bleaching of DPPH was measured at 517 nm. The DPPH radical scavenging activity was calculated by the formula described by Salta et al. (2010).

### Statistical Analysis

All measurements were carried out in three biological replicates. Data were expressed as the mean ± standard deviation. A two-way ANOVA analysis for treatment and time of storage was performed. When the effect of treatment was compared with control, a one-way ANOVA analysis for the treatment effect as run at each time of storage. The mean values were separated using the Tukey HSD test (*p* < 0.05). The data were analyzed with the SPSS 17.0 software (SPSS Inc., Chicago, IL, USA).

## Results

### Influences of Ethylene Treatment on Peel Browning of ‘Huangguan’ Pear

**FIGURE 1:**
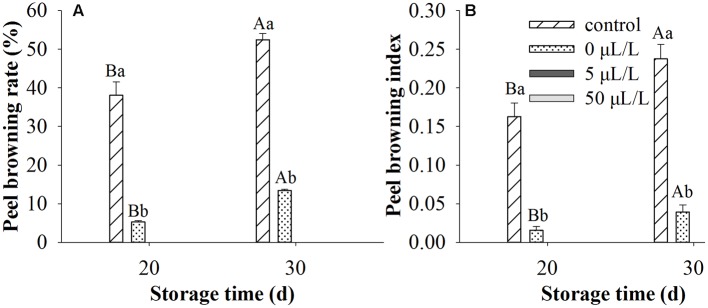
**The incidence of brown spot disorder in ‘Huangguan’ pears treated with ethylene.** **(A)** Peel browning rate (%); **(B)** Peel browning index. Fruits with rapid cooling at 0°C after harvest served as control. Fruits were first treated with various concentrations of ethylene at 20°C for 8 h and then stored at 0°C for evaluations. Bars marked by the same capital letter or lowercase letter indicate that values were not statistically different among sampling days for the same treatment or among treatments for the same sampling day, respectively (*p* > 0.05).

Though ethylene was widely used for the ripening of fruits, the present research studied the effects of ethylene treatments on peel browning inhibition of ‘Huangguan’ pear. We first examined whether ethylene treatments affect fruit quality. Compared with control, ethylene treatments at 50 μL/L exhibited no significant influence on TA, TSS and firmness of fruits stored at 0°C for 100 and 200 days (**Supplementary Table S1**). Ethylene treatments at different concentrations (0, 5, and 50 μL/L) were performed for investigating its effect on skin browning of ‘Huangguan’ pear (**Figure 1**). We found that the peel browning mainly occurred at the early storage stage (data not shown), which is consistent with the investigations of many postharvest industries in the production area. Thereby, the browning rate and browning index were examined only on day 20 and 30 post storage. Peel browning index and rate were significantly affected by treatment, storage time and their interaction. Therefore the single effects were reported in **Figure 1**. The results showed that ethylene at various concentrations significantly reduced the browning compared with control when pears were stored at 0°C for 20 and 30 days. Ethylene treatments at 5 and 50 μL/L prevented the browning disorder during 200 days storage (data not shown). Interestingly, compared with control (rapid cooling at 0°C after harvest), 0 μL/L treatment (fruits were first placed at 20°C for 8 h and then stored at 0°C) also reduced the disorder.

As shown in **Table 1**, the browning index of pears stored at 0°C (rapid cooling) for 20 days reached 0.30. However, stored at 20°C for 8 h prior to cold storage (slow cooling) significantly decreased the browning index to 0.17. Nevertheless, this browning inhibition effect was no longer significant 30 days post storage at 0°C, compared to control. Though slow cooling significantly decreased the browning index, no differences were found in browning rate, indicating that slow cooling delayed the onset of browning, not the occurrence. 1-MCP slightly reduced the browning rate and index, however, no significant difference was existed when compared with control. Ethylene dramatically decreased the incidence of browning. The browning rates were only 6.80 and 16.59% after storage for 20 and 30 days, respectively. When ethylene was applied with 1-MCP, its inhibition efficiency was no longer remarkable (**Table 1** and **Supplementary Figure S1**).

**Table 1:** Effect of ethylene and 1-MCP on peel browning rate and index of ‘Huangguan’ pear.

	Peel browning rate		Peel browning index	
		
	20 days	30 days	20 days	30 days
Control	73.80 ± 9.28^a^	86.92 ± 7.78^a^	0.30 ± 0.02^a^	0.48 ± 0.05^a^
Air	49.48 ± 13.51^a^	82.08 ± 15.8^a^	0.17 ± 0.07^b^	0.37 ± 0.12^a^
1-MCP	54.77 ± 2.83^a^	73.91 ± 6.52^a^	0.18 ± 0.02^ab^	0.32 ± 0.08^a^
Ethylene	6.80 ± 2.18^b^	16.59 ± 5.33^b^	0.02 ± 0.01^c^	0.04 ± 0.01^b^
1-MCP + Ethylene	64.52 ± 11.8^a^	83.70 ± 15.13^a^	0.24 ± 0.09^ab^	0.45 ± 0.06^a^


*Control: fruits were rapidly cooled at 0°C; Air: fruits were first placed at 20°C for 8 h, then held at 0°C; 1-MCP: fruits were first treated with 1-MCP at 20°C for 8 h, then held at 0°C; Ethylene: fruits were first treated with ethylene (ethephon sachet) at 20°C for 8 h, then held at 0°C; 1-MCP + ethylene: fruits were first treated with 1-MCP and ethylene at 20°C for 8 h, then held at 0°C. During the 8 h treatment, the concentrations of ethylene and 1-MCP were detected at 0.70–1.28 and 1 μL/L, respectively. Data are expressed as mean ± SD (*n* = 3). Values in a column marked by the same letter were not statistically different (*p* > 0.05).*

**FIGURE 2:**
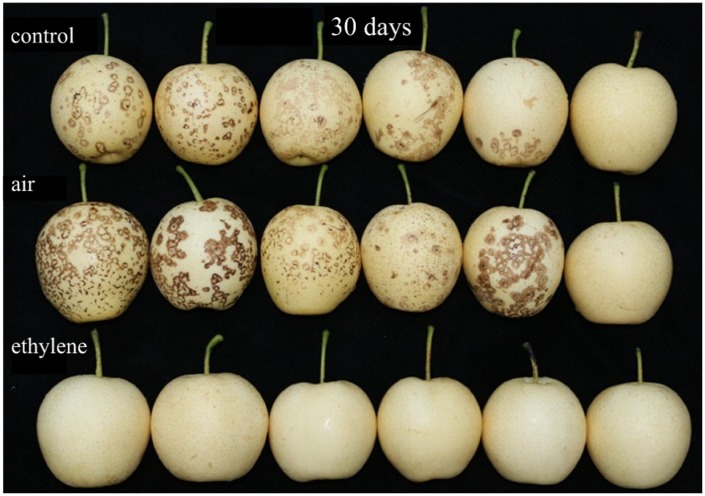
**Effect of ethylene on the incidence of spots brown in ‘Huangguan’ pear after 30 days storage at 0°C.** Fruits with rapid cooling at 0°C after harvest served as control. Fruits were treated with air or ethylene (5 μL/L) at 20°C for 8 h and then stored at 0°C for analysis.

The effect of ethylene treatment on peel browning was verified (**Figure 2**). The results showed that ethylene treatment at 5 μL/L significantly inhibited the browning. Thereby, ethylene concentration at 5 μL/L was used in the following experiment to investigate the physiological functions of ethylene on ‘Huangguan’ pear.

### Membrane Permeability

**FIGURE 3:**
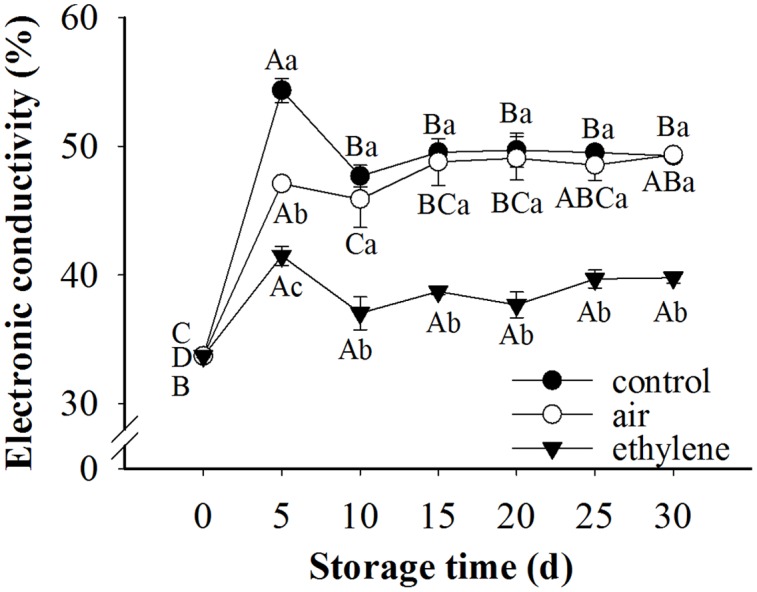
**Effect of ethylene on the electrolyte leakage rate of ‘Huangguan’ pear peel.** Fruits with rapid cooling at 0°C after harvest were served as control. Fruits were first treated with air and ethylene (5 μL/L) at 20°C for 8 h and then stored at 0°C for evaluations. Values marked by the same capital letter or lowercase letter indicate that values were not statistically different among sampling days for the same treatment or among treatments for the same sampling day, respectively (*p* > 0.05).

To understand the physiological bases for the ethylene inhibition effects on the peel browning, we first measured electrolyte leakage, an indicator of the change of pear peel fruit in membrane permeability. Given that the effect of treatment, storage time as well as their interaction were significant for membrane permeability, therefore the single effects were reported in **Figure 3**. Electrolyte leakage in the control and treatments was sharply increased during the first 5 days of storage and then slightly decreased, which was still higher than 0 day (**Figure 3**). After 10 days storage, no significant difference was found between the control and air treatment in the electrolyte leakage. However, the electrolyte leakage in the pear fruit treated with ethylene changed slowly during the whole storage period. The difference in electronic conductivity among treatments after 5 days storage was attributed to the difference in treatment since the effect of storage time on electronic conductivity was slight. Compared with control and air treated fruits, ethylene-treated fruits exhibited much lower electronic conductivity, indicating that the electrolyte leakage caused by the disruption of membrane permeability was dramatically prevented by ethylene.

### Respiration Rate of Pear Fruit

**FIGURE 4:**
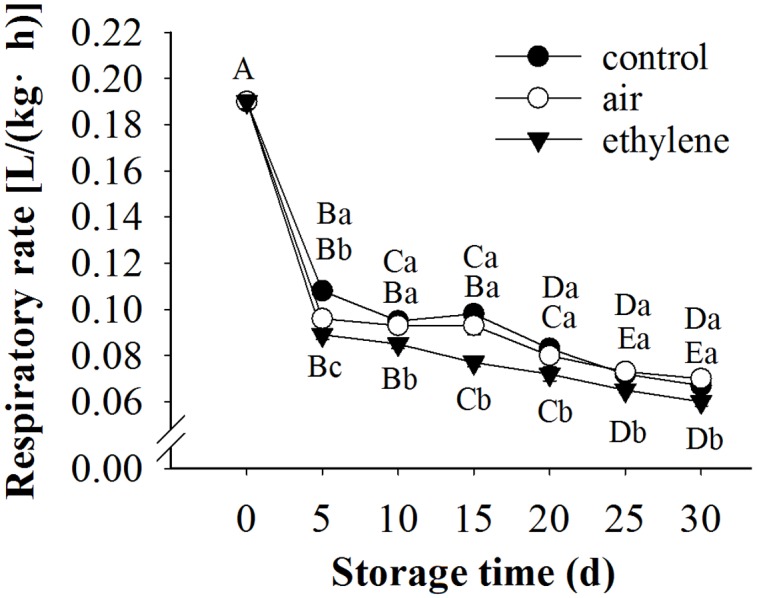
**Effect of ethylene on the respiration rate of ‘Huangguan’ pear.** Fruits with rapid cooling at 0°C after harvest were served as control. Fruits were first treated with air and ethylene (5 μL/L) at 20°C for 8 h and then stored at 0°C for analysis. Values marked by the same capital letter or lowercase letter indicate that values were not statistically different among sampling days for the same treatment or among treatments for the same sampling day, respectively (*p* > 0.05).

The respiration rate of fruits was sharply decreased during the first 5 days of storage, and the decreasing trend slowed afterward (**Figure 4**). The respiration rate was significantly affected by treatment and storage time as well as their interaction. Ethylene application significantly inhibited the respiration rate, compared to the control and air treatment.

### Total Phenolics Content and PPO Activity

**FIGURE 5:**
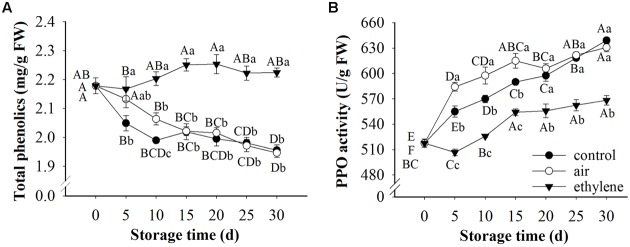
**Effects of ethylene on pear phenolic metabolism during the storage at 0°C.** **(A)** Total phenolic content; **(B)** PPO activity. Fruits with rapid cooling at 0°C after harvest were served as control. Fruits were first treated with air and ethylene (5 μL/L) at 20°C for 8 h and then stored at 0°C for analysis. Values marked by the same capital letter or lowercase letter indicate that values were not statistically different among sampling days for the same treatment or among treatments for the same sampling day, respectively (*p* > 0.05).

Total phenolics content and PPO activity in pears during cold storage were significantly affected by treatment, storage time and their interaction (**Figure 5**). Significant differences were found between ethylene-treated and non-ethylene-treated pears in total phenolics content and PPO activity, except for total phenolics content in air-treated pears at day 5. Compared with control, ethylene at 0 μL/L showed no effect on preventing the loss of phenolics, except for day 10. Ethylene maintained higher content of phenolics during the 30 days storage, whereas the rapid cooling control and 0 μL/L ethylene (air) decreased the contents. Fruits in all three groups exhibited similar patterns in PPO activity, which was increased during the whole storage time. However, ethylene treatments significantly prevented the increase of PPO activity (**Figure 5**).

### POD, CAT, APX and SOD Activities

**FIGURE 6:**
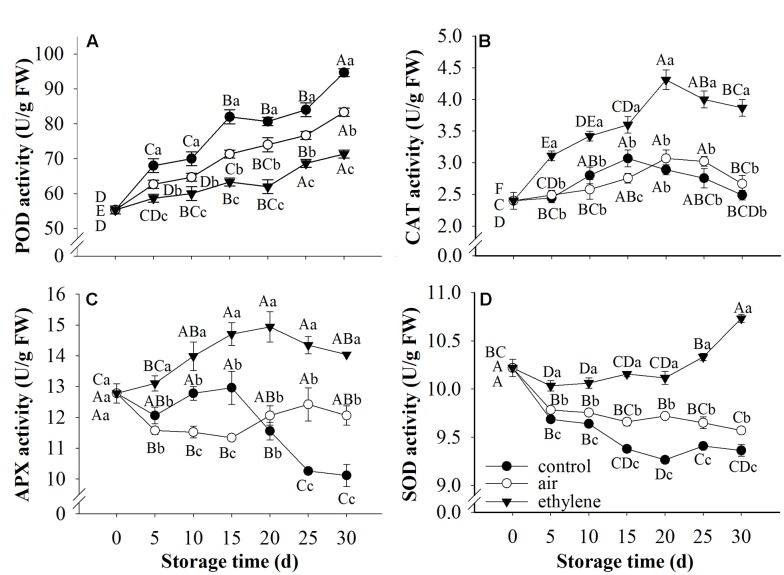
**Effects of ethylene on the activities of antioxidant enzymes.** **(A)** POD activity; **(B)** CAT activity; **(C)** APX activity; **(D)** SOD activity. Fruits with rapid cooling at 0°C after harvest were served as control. Fruits were first treated with air and ethylene (5 μL/L) at 20°C for 8 h and then stored at 0°C for analysis. Values marked by the same capital letter or lowercase letter indicate that values were not statistically different among sampling days for the same treatment or among treatments for the same sampling day, respectively (*p* > 0.05).

Peroxidase, CAT, APX and SOD are considered as antioxidant enzymes, participate in the defense of fruit against stresses through scavenging free radicals. The ANOVA result showed that treatment, storage time and their interaction were significant for POD, CAT and APX as well as SOD. The changes in the activity of these enzymes in fruits treated with ethylene and air were demonstrated in **Figure 6**. Peroxidase activity in all samples was increased during cold storage. However, ethylene treatment showed strong inhibition on the increase of POD activity (**Figure 6A**). Catalase activity was increased at the early stage of storage and then decreased thereafter. Fruits without ethylene treatment (rapid cooling) and treated with air (slow cooling) only showed distinctively lower levels of CAT activity than fruits treated with 5 μL/L ethylene (**Figure 6B**). Ascorbate peroxidase activity in the fruits treated with ethylene was increased at the early stage of storage and then slightly decrease (**Figure 6C**), and was remarkably higher than that in control and air-treated fruits. APX activity in untreated fruits was stable during the first 15 days storage and dramatically decreased thereafter. Ethylene treatments resulted in the induction of SOD activity (**Figure 6D**). In general, SOD activity in ethylene-treated fruit peel was remarkably higher than that in the control and air-treated fruit. Interestingly, SOD activity in air-treated fruits was also significantly higher than the control (*p* < 0.05).

### DPPH Radical Scavenging Activity

**FIGURE 7:**
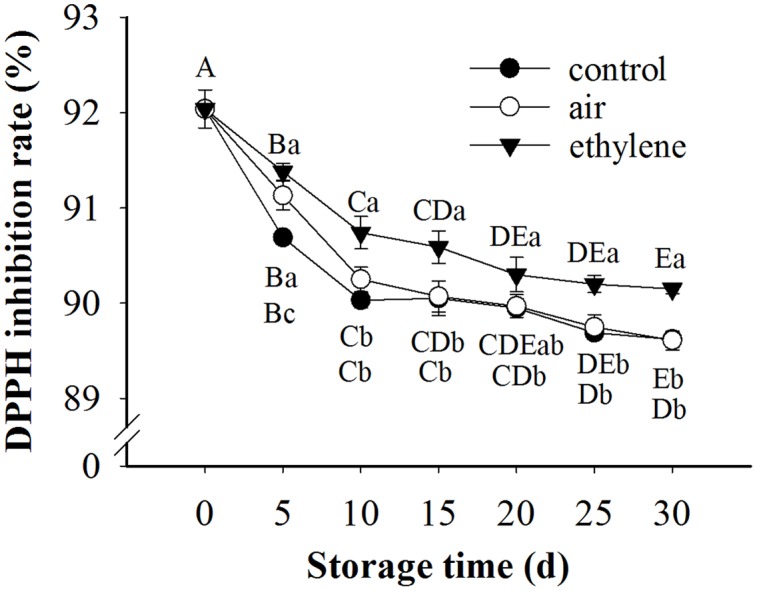
**Effect of ethylene on DPPH scavenging activity in ‘Huangguan’ pear.** Fruits with rapid cooling at 0°C after harvest were served as control. Fruits were first treated with air and ethylene (5 μL/L) at 20°C for 8 h and then stored at 0°C for analysis. Values marked by the same capital letter or lowercase letter indicate that values were not statistically different among sampling days for the same treatment or among treatments for the same sampling day, respectively (*p* > 0.05).

The radical scavenging activity in the pear samples was decreased with the extension of storage (**Figure 7**). During the whole storage, the radical scavenging activity in the ethylene-treated fruits was over 90%, and remarkably higher than the control and air treatment. After 5 days storage, no significant difference in radical scavenging activity was observed between the control and air treatment (*p* > 0.05).

## Discussion

As a commonly occurred physiological disorder in fruits and vegetables, browning disorder has been heavily studied (Cantos et al., 2002; Franck et al., 2007; Chung and Moon, 2009; Gong et al., 2010). The present study demonstrated that postharvest “Huangguan” pear fruits were susceptible to brown spot disorder a few days after storage at 0°C (**Table 1** and **Figure 1**). Though ethylene was believed to be responsible for the ripening and senescence of fruits, the present study revealed that application of exogenous ethylene inhibited the peel browning of ‘Huangguan’ pear. Most of all, compared with control, no change in edible quality (TA, TSS and firmness) in the fruits treated with ethylene were observed. Overall, ethylene showed no negative influence on the edible quality (**Supplementary Table S1**). Similarly, Bower et al. (2003) reported that after 3-month cold storage and 4 days ripening at 20°C, pears treated with various concentrations of ethylene exhibited almost the same firmness to control.

Ethylene at the concentrations of 5 and 50 μL/L effectively prevented the browning disorder of fruits during 200 days storage (data not shown). Compared with control (rapid cooling at 0°C after harvest), 0 μL/L treatment also decreased the disorder. The inhibiting effect of 0 μL/L treatment may be due to delay cooling, since fruits treated with ethylene were placed at 20°C for 8 h before being stored at 0°C. Our previous research showed that delayed cooling can reduce the browning disorder of ‘Huangguan’ pear, though the effects are limited (Wang and Wang, 2011).

The inhibition of 1-MCP on the browning of fruits is widely reported (Argenta et al., 2003; Fu et al., 2007). The core browning of 1-MCP treated ‘Yali’ pear was reduced by 91% after 100 days storage (Fu et al., 2007). They assumed that the beneficial effect of 1-MCP on reducing physiological disorder may be attributed to the increase in antioxidant potential as well as the inhibition of ethylene production and respiration rate. However, this study showed that the browning inhibition of 1-MCP on ‘Huangguan’ pear was not significant (**Table 1** and **Supplementary Figure S1**). The differences between studies may be due to the differences in pear varieties and the concentration of 1-MCP applied, since it was reported that the 1-MCP-induced response was dose-dependent (Argenta et al., 2003).

Interestingly, our data demonstrated that the presence of ethylene inhibited the occurrence of peel browning of ‘Huangguan’ pear up to 200 days of storage (**Supplementary Figure S1**). However, it was reported that low levels of ethylene (0.01 and 1 μL/L) aggravated the flesh and core browning of Japanese pears (Szczerbanik et al., 2007). The contradiction between studies may result from the amounts and duration of ethylene as well as the pear cultivars used. In the present study, ‘Huangguan’ pears were treated by 5 or 50 μL/L ethylene for 8 h before cold storage, whereas Szczerbanik et al. (2007) treated ‘Nijisseiki’ pear with <0.005, 0.01, 0.1 and 1 μL/L ethylene for 26 weeks. Moreover, the incidence of physiological disorder (such as browning) of ‘Bartlett’ pear was increased after treated by ethylene (0, 1, 5 and 10 μL/L) for 3 months (Bower et al., 2003). These results indicated that when ethylene was used for browning inhibition, the treatment time should be well controlled, otherwise adverse results may be observed.

Due to its competitive binding to ethylene receptors with ethylene, 1-MCP was widely used to alleviate the ripening and senescence of fruits caused by ethylene (Argenta et al., 2003; Liu et al., 2013). It is worth noting that the browning inhibition effect of ethylene was eliminated when ethylene was applied with 1-MCP at the same time (**Table 1** and **Supplementary Figure S1**). This may be attributed to the competitive binding of 1-MCP to ethylene receptor, which affected the transduction of ethylene signal and thereby influenced the response of ethylene (Morgan and Drew, 1997).

Membrane integrity plays an important role in prevention of the occurrence of browning, since it separates the substrates from enzymes (Kou et al., 2015). Moreover, Duan et al. (2011) demonstrated that the electrolyte leakage was alleviated while the compartmentation of enzymes and substrate was maintained by pure oxygen treatment. Electrolyte leakage rate is considered as an indirect measure of membrane damage (Liu and Wang, 2012). The higher the electrolyte leakage rate is, the more the membrane damage. The disruption of membrane integrity was associated with the browning of mushroom (Liu and Wang, 2012). Also, Cantos et al. (2002) found that maintaining membrane integrity was potentially an important approach to control browning. In the present research, the electronic conductivity of fruits was significantly increased after 5 days storage at 0°C. This may be attributed to the stress response of fruits to cold storage, since ‘Huangguan’ pear is very sensitive to cold. Ethylene treatment obviously restrained the increase of the electrolyte leakage rate and maintained the integrity of the cell membrane, (**Figures 1**–**3**). The lower electronic conductivity of ethylene treated fruits indicated the improved defense capacity of fruits to stresses. These results revealed that ethylene increased the defense capacity of fruits and thereby inhibited the peel browning which may be caused by chilling injury. This was consistent with previous reports that ethylene was involved in chilling injury and recovery of fruits (Yang and Hoffman, 1984; Morgan and Drew, 1997).

Higher respiration rate triggers a faster overall deterioration and metabolic activity (Chung and Moon, 2009). Endogenous or exogenous ethylene is usually considered to be associated with high respiration rate. However, in the present study, the respiration rate of fruits treated with ethylene was significantly lower than that in the control and air-treated fruits (**Figure 4**). This may be attributed to the effect of ethylene to the internal gas partial pressure, which could influence the respiration rate and the antioxidant system (Franck et al., 2007). Moreover, it was reported that application of ethylene to pears increased the internal level of ethylene and CO_2_, but decreased the level of O_2_ (Szczerbanik et al., 2007).

Phenolics and PPO are believed to be associated with enzymatic browning. Ethylene treatment maintained the high content of phenolics and inhibited the activity of PPO, thereby preventing the incurrence of browning. Similarly, Kou et al. (2015) reported that pear browning was inhibited by 2% CaCl_2_ or 1% pullulan, which delayed the degradation of phenolics and inhibited PPO activity. The higher content of phenolics in ethylene treated fruits may be due to the induction of ethylene to PAL, which is the initial rate controlling enzyme in phenolic synthesis (Ke and Saltveit, 1989). To some extent, ethylene induced the defense response of fruits to stress, leading to the synthesis of phenolics. Previous research also found that exposure lettuce to 10 μL/L ethylene induced the production of individual phenolic compounds (Tomás-Barberán et al., 1997). The inhibition of ethylene on PPO activity may be due to its influence on Cu^2+^, which is contained in the active center of PPO (Kou et al., 2015). However, further study needs to be done to reveal the mechanism of ethylene inhibiting PPO activity. Pear fruits treated with ethylene exhibited higher DPPH radical scavenging activity than the control and air treatment. The higher antioxidant activity may be due to the higher content of phenolics in ethylene-treated fruits, since the phenolics content was strongly correlated with antioxidant activity (Malenèič et al., 2007; Ma and Huang, 2014). Moreover, the antioxidant capacity of banana fruit was promoted by the accumulation of phenolic compounds (Wang et al., 2014).

Under normal circumstances, reactive oxygen species (ROS) are produced during cellular functional activity and participate in cellular metabolism (Lü et al., 2010). Usually, the organisms scavenge excessive radicals and keep the balance of ROS in cells through the enzymatic and non-enzymatic antioxidant systems. However, under various stresses, ROS may accumulate, resulting in the breaking of the balance and inducing the occurrence of physiological disorders (Valko et al., 2006; Ma et al., 2014). The accumulation of ROS may lead to the peroxidation of membrane lipid, thereby disrupting the membrane integrity and, resulting in the occurrence of browning. Antioxidant enzymes such as POD, SOD, CAT and APX play important roles in scavenging ROS. SOD catalyzes the superoxide radical to H_2_O_2_ while POD, CAT and APX responsible for the elimination of H_2_O_2_ (Duan et al., 2011; Liu et al., 2013). In this study, severe browning was observed in pear fruits without treatment. Due to the sensitivity of ‘Huangguan’ pear to cold, the peel browning of fruits may be caused by chilling, which is a severe stress for fruits. Accompanied with browning, the activity of SOD and APX were decreased with storage, while CAT was first increased and followed by a slight decrease (**Figure 6**). Results from the electronic conductivity showed that the membrane integrity was disrupted since the electronic conductivity significantly increased after 5 days storage (**Figure 3**). All together, these results indicated that browning of ‘Huangguan’ pear resulted from the accumulation of ROS and the decreasing activity of antioxidant enzymes. This is in agreement with previous studies that the membrane lipid peroxidation induced by the decreased scavenging activity and accumulation of free radicals contributes to the skin browning of ‘Huangguan’ pear (Kou et al., 2015). Results from this study demonstrated that ethylene treatment maintained the high activity of SOD, CAT and APX, stabilized the membrane integrity and eventually prevented the peel browning of ‘Huangguan’ pear fruits. We concluded that the browning inhibition of ethylene on ‘Huangguan’ pear was achieved by improving the antioxidant defense systems of fruits. Similarly, studies reported that exposure to pure oxygen induced the activities of SOD, CAT and APX, alleviated the lipid peroxidation and kept membrane integrity, thereby inhibited the browning of litchi fruit (Duan et al., 2011; Liu et al., 2013). Moreover, the inhibition of browning is concomitant with higher phenolic compounds content, lower PPO activity, and higher CAT and SOD activities (Kou et al., 2015). Interestingly, the similarity between ethylene and pure oxygen treatments was that they both improved the enzymatic antioxidant defense system of fruits by affecting the activity of antioxidant enzymes (SOD, APX and CAT). Pure oxygen treatment may promote ethylene production through enhancing of ACC oxidase activity, implying that they may share a common mode of action via ethylene pathway.

## Conclusion

Ethylene treatment enhanced the antioxidant defense capacity of fruits to stress. Fruits treated by ethylene exhibited higher phenolic content and DPPH radical scavenging activity, higher activity of antioxidant enzymes (SOD, CAT and APX) and lower PPO activity and electrolyte leakage rate. These factors were evidently responsible for the lower browning incidence of ethylene-treated ‘Huangguan’ pears.

## Author Contributions

QW and C-ZJ conceived the concept. MY, JW, and YM performed the experiments and data analyses. YM, QW, and C-ZJ wrote the manuscript. C-ZJ extensively revised the manuscript. All authors read and approved the manuscript.

## Conflict of Interest Statement

The authors declare that the research was conducted in the absence of any commercial or financial relationships that could be construed as a potential conflict of interest.
