# Supplementary material for: Application of Exogenous Ethylene Inhibits Postharvest Peel Browning of ‘Huangguan’ Pear
Source: Front Plant Sci. 2017 Jan 18;7:2029. doi: 10.3389/fpls.2016.02029 (PMC5241572; doi:10.3389/fpls.2016.02029)
Supplement: Supplementary file 1 [file Table_1.pdf]

Supplementary Material

Application of Exogenous Ethylene Inhibits Postharvest Peel Browning of 'Huangguan' Pear

Yurong Ma, Mengnan Yang, Jingjing Wang, Cai-Zhong Jiang* and Qingguo Wang*

*** Correspondence:** Corresponding Author: Cai-Zhong Jiang, cjiang@ucdavis.edu; Qingguo Wang, wqgyyy@126.com

1 Supplementary Table

Supplementary Table 1. Effects of ethylene (50 μL/L) on titratable acidity (TA), total soluble solids (TSS) and firmness of 'Huangguan' pear flesh

	Storage time (d)		
	0	100	200
TA (%)			
control	0.164 ± 0.004	0.123 ± 0.006^{a}	0.114 ± 0.007^{a}
air		0.120 ± 0.003^{a}	0.112 ± 0.003^{a}
ethylene		0.129 ± 0.005^{a}	0.122 ± 0.007^{a}
TSS (%)			
control	11.17 ± 0.15	11.73 ± 0.33^{a}	10.93 ± 0.23^{b}
air		11.83 ± 0.25^{a}	11.23 ± 0.23^{ab}
ethylene		12.27 ± 0.31^{a}	11.47 ± 0.21^{a}
Firmness (kg/cm^2)			
control	3.61 ± 0.18	3.31 ± 0.02^{ab}	3.15 ± 0.06^{a}
air		3.27 ± 0.06^{b}	3.17 ± 0.10^{a}
ethylene		3.43 ± 0.08^{a}	3.21 ± 0.04^{a}

Control: fruits were rapidly cooled at 0°C; air: fruits were first placed at 20°C for 8 h, then held at 0°C; ethylene: fruits were first treated with ethylene (5 μL/L) at 20°C for 8 h, then held at 0°C. Data are expressed as mean ± SD (n = 3). Values in a column marked by the same letter were not statistically different (p> 0.05).

To examine long-term effects of ethylene treatments on the fruit quality, the firmness, TA and TSC were measured using the pears harvested in 2013. The pears were randomly divided into 3 groups, including the control, 0 and 50 μL/L ethylene treatment (18 fruits/replicate and 3 replicates/group. After treatment, pears were packed with foam sleeves, placed into trays wrapped with plastic film and stored at 0℃ with RH 70%~80%. Samplings were taken at storage days of 0, 100 and 200.

For TA, TSS and firmness analysis, 6 fruits were randomly picked from each replicate. For TA measurement, 5 g of fruit pulp were used and extracted with distilled water (25 mL), for 30 min. Then the mixture was filtered and 10 mL of the solution was titrated with 0.01

mol/L NaOH to pH 8.2. TA was expressed as percent malic acid. Fruit juice was collected onto a digital refractometer (ATAGO PAL-1, Tokyo, Japan) for TSS measurement of Brix. Flesh firmness was measured at the equator on four side of the skin-removed fruit, using a hand-held penetrometer (Wanger Instruments, US) with 8 mm plunger diameter. Firmness was the mean value of four independent readings for each pear and expressed as kg/cm^2.
