# Supplementary material for: Application of Exogenous Ethylene Inhibits Postharvest Peel Browning of ‘Huangguan’ Pear
Source: Front Plant Sci. 2017 Jan 18;7:2029. doi: 10.3389/fpls.2016.02029 (PMC5241572; doi:10.3389/fpls.2016.02029)
Supplement: Supplementary file 2 [file Image_1.pdf]

Supplementary Material

Application of Exogenous Ethylene Inhibits Postharvest Peel Browning of 'Huangguan' Pear

Yurong Ma, Mengnan Yang, Jingjing Wang, Cai-Zhong Jiang* and Qingguo Wang*

*** Correspondence:** Corresponding Author: Caizhong Jiang, cjiang@ucdavis.edu; Qingguo Wang, wqgyyy@126.com

1 Supplementary Figure

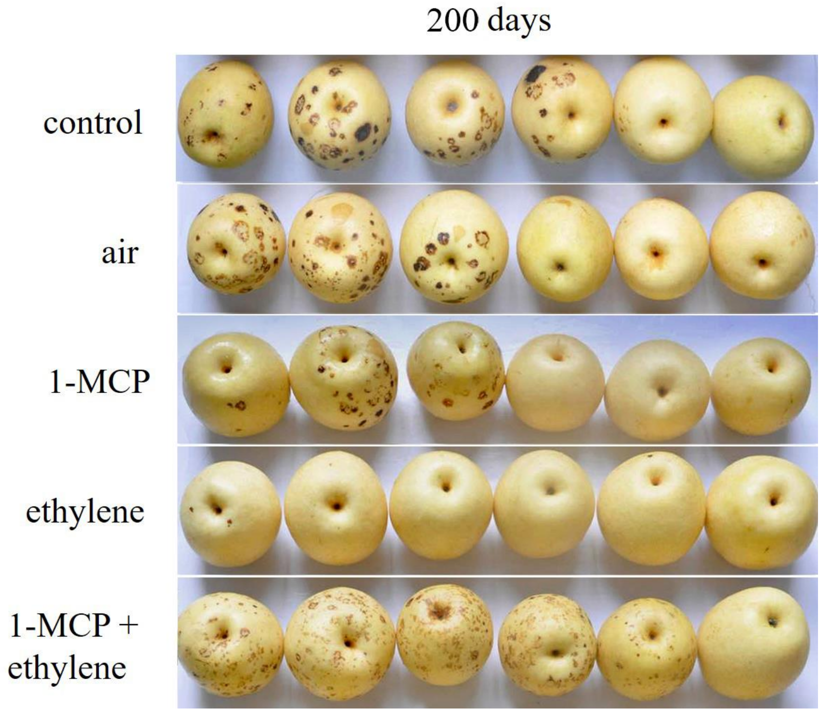

Supplementary Figure 1. Effect of ethylene and 1-MCP on the incidence of spots brown in 'Huangguan' pear after storage at 0°C for 200 d

Control: fruits were rapidly cooled at 0°C;

Air: fruits were first placed at 20°C for 8 h, then held at 0°C;

1-MCP: fruits were first treated with 1-MCP (1-MCP cyclodextrin sachet) at 20°C for 8 h, then held at 0°C;

Ethylene: fruits were first treated with ethylene (ethephon sachet) at 20°C for 8 h, then held at 0°C;

1-MCP + ethylene: fruits were first treated with 1-MCP (1-MCP cyclodextrin sachet) and ethylene (ethephon sachet) at 20°C for 8 h, then held at 0°C.

During the 8 h treatment, the concentrations of ethylene and 1-MCP were detected at 0.7028 µL/L and 1 µL/L, respectively.
